# Supplementary material for: Higher fall rates and broader kinematic diversity in bilateral versus unilateral unconstrained slips
Source: PLoS One. 2025 Aug 7;20(8):e0328900. doi: 10.1371/journal.pone.0328900 (PMC12331078; doi:10.1371/journal.pone.0328900)
Supplement: S1 Table — (PDF) [file pone.0328900.s002.pdf]

Table 3.2. Description of the Unilateral and Bilateral Slips' Probabilistic Graphical Models (PGMs) Combined in Both Sagittal & Frontal Planes

Trigger	1. Slip Initiation	Description	2. Trailing Leg Touchdown	Description	3. Mean Feet Velocities 100ms Post Trailing Leg Touchdown	Description	Outcome
Unilateral (n=30) or Bilateral (n=28) Slips triggered at Leading Leg's Early Stance	1.1) Trailing in anterior motion frontally arrested, Leading slide anterior and contralateral (n=5)	a) Slip Initiation Onset = Velocities were extracted within 30ms of slip start (see text) and visually confirmed to verify feet's motions due to slips before H-reflex latencies.	2.1) Trailing foot Touchdown Posterior (Sagittal) & Lateral (Frontal) to CoM (n=24)	a) Trailing Leg Touchdown Onset = Onsets were confirmed by CoP and GRFs change due to impact	3.1) Trailing Arrested, Leading Arrested (n=1)	a) Equation: $\bar{v} = \frac{\Delta \vec{x}}{100ms}$ where $\bar{v}$ is average velocity, $\Delta \vec{x}$ is the displacement.	Recovery = Load cell force attached to the harness < 4.51% of Body-weight across 1 second (n=49)
	1.2) Trailing in anterior and lateral motion, Leading Slide anterior and contralateral (n=17)	b) Foot in Motion = Slip Velocity ≥±0.1ms	2.2) Trailing foot Touchdown Under (Sagittal) & Lateral (Frontal) to CoM (n=22)	b) Touchdown posterior to COM (Sagittal) = CoM's projection is anterior to the Toe's marker	3.2) Trailing in anterior and contralateral motion, Leading in anterior motion frontally arrested (n=7)	b) Foot in Motion = Slip Velocity ≥±0.1ms	Harness-Assisted Recovery = Load cell force attached to the harness >10.28% of Body weight across 0.2 second and <30% of Body-weight (n=3)
	1.3) Trailing in anterior and lateral motion, Leading slide anterior frontally arrested (n=12)	c) Foot Arrested = Slip Velocity ≤±0.099ms	2.3) Trailing leg Touchdown Anterior (Sagittal) & Lateral (Frontal) to CoM (n=10)	c) Touchdown under the COM (Sagittal) = CoM's projection is inside to the foot's boundaries (i.e., within any foot marker)	3.3) Trailing in anterior and contralateral motion, Leading arrested (n=3)	c) Foot Arrested = Slip Velocity ≤±0.099ms	Fall = Peak load cell force attached to the harness >30% of Body-weight (n=6)
	1.4) Trailing in posterior and lateral motion, Leading Slides anterior and contralateral (n=8)	d) Anterior Motion = Positive Foot sagittal velocity. The foot in forward motion relative to the CoM	2.4) Trailing leg Touchdown Anterior (Sagittal) & Under (Frontal) to CoM (n=2)	d) Touchdown anterior to COM (Sagittal) = CoM's projection is posterior to the Heel's marker	3.4) Trailing in anterior and lateral motion, Leading in anterior motion frontally arrested (n=4)	d) Anterior Motion = Positive Foot sagittal velocity. The foot in forward motion relative to the CoM.	
	1.5) Trailing in posterior and contralateral motion, Leading Slides anterior and contralateral (n=3)	e) Posterior Motion = Negative Foot sagittal velocity. The foot in backward motion relative to the CoM		e) Touchdown lateral to COM (Frontal) = CoM's projection is contralateral (inward) to the medial marker (R/LCA2)	3.5) Trailing in anterior motion frontally arrested, Leading in anterior motion frontally arrested (n=3)	e) Posterior Motion = Negative Foot sagittal velocity. The foot in backward motion relative to the CoM.	
	1.6) Trailing in anterior motion frontally arrested, Leading slide anterior frontally arrested (n=8)	f) Lateral Motion = Positive Foot frontal velocity. The foot in outward motion relative to the CoM (e.g., rightward in case of the right leg and vice versa).		f) Touchdown under the COM (Frontal) = CoM's projection is inside to the foot's boundaries (i.e., within any foot marker)	3.6) Trailing in anterior motion frontally arrested, Leading in anterior and lateral motion (n=11)	f) Lateral Motion = Positive Foot frontal velocity. The foot in outward motion relative to the CoM (e.g., rightward in case of the right leg and vice versa).	
	1.7) Trailing in posterior motion frontally arrested, Leading slide anterior and contralateral (n=4)	g) Contralateral Motion = Negative Foot frontal velocity. The foot in inward motion relative to the CoM (leftward in case of the right leg and vice versa).			3.7) Trailing in anterior and lateral motion, Leading in lateral motion sagittally arrested (n=1)	g) Contralateral Motion = Negative Foot frontal velocity. The foot in inward motion relative to the CoM (leftward in case of the right leg and vice versa).	
	1.8) Trailing in anterior and contralateral motion, Leading Slides anterior and lateral (n=1)				3.8) Trailing in anterior motion frontally arrested, Leading in anterior and contralateral motion (n=1)		
					3.9) Trailing in contralateral motion sagittally arrested, Leading in anterior and lateral motion(n=1)		
					3.10) Trailing in anterior and contralateral motion, Leading in anterior and lateral motion (n=19)		
					3.11) Trailing in anterior and lateral motion, Leading in anterior and lateral motion (n=7)		

Descriptions of feet positions and velocities are relative to the CoM. Slip velocity = Raw feet velocity.
