# Supplementary material for: Higher fall rates and broader kinematic diversity in bilateral versus unilateral unconstrained slips
Source: PLoS One. 2025 Aug 7;20(8):e0328900. doi: 10.1371/journal.pone.0328900 (PMC12331078; doi:10.1371/journal.pone.0328900)
Supplement: S2 Dataset — (PDF) [file pone.0328900.s003.pdf]

Slip Entropy Calculation

This code uses a probabilistic graphical model representation of slip uncertainty and calculates the entropy of the model to characterize slip diversity.

Load slip state transition probabilities

```
probs_u = readtable('Markov_Chains_Probabilities_7Sep22.xlsx',...
    'Sheet', 'Probabilities-Unil');
```

```
Warning: Column headers from the file were modified to make them valid MATLAB identifiers before creating
variable names for the table. The original column headers are saved in the VariableDescriptions property.
Set 'VariableNamingRule' to 'preserve' to use the original column headers as table variable names.
```

```
probs_b = readtable('Markov_Chains_Probabilities_7Sep22.xlsx',...
    'Sheet', 'Probabilities-bil');
```

```
Warning: Column headers from the file were modified to make them valid MATLAB identifiers before creating
variable names for the table. The original column headers are saved in the VariableDescriptions property.
Set 'VariableNamingRule' to 'preserve' to use the original column headers as table variable names.
```

Get the Four Transition Probability Matrices for Unilateral Slips

```
% 1: Trigger (TR) to (SI)
T_TR_SI_u = probs_u{1,3:10};
T_TR_SI_u = convertTransMat(T_TR_SI_u)
```

```
T_TR_SI_u = 1×8
    0.1667    0.4667    0.3000         0         0    0.0667         0         0
```

```
T_TR_SI_b = probs_b{1,3:10};
T_TR_SI_b = convertTransMat(T_TR_SI_b)
```

```
T_TR_SI_b = 1×8
         0    0.1786    0.0357    0.2857    0.1071    0.2143    0.1429    0.0357
```

```
% 2: SI to Trailing Leg Touchdown (TLTD)
T_SI_TLTD_u = probs_u{2:9, 11:14};
T_SI_TLTD_u = convertTransMat(T_SI_TLTD_u)
```

```
T_SI_TLTD_u = 8×4
    0.8000    0.2000         0         0
    0.5000    0.2143    0.1429    0.1429
    0.3333    0.4444    0.2222         0
         0         0         0         0
         0         0         0         0
    0.5000    0.5000         0         0
         0         0         0         0
         0         0         0         0
```

```
T_SI_TLTD_b = probs_b{2:9, 11:14};
T_SI_TLTD_b = convertTransMat(T_SI_TLTD_b)
```

```
T_SI_TLTD_b = 8×4
         0         0         0         0
         0    0.4000    0.6000         0
         0         0    1.0000         0
    0.8750    0.1250         0         0
    0.3333    0.6667         0         0
         0    0.6667    0.3333         0
    0.2500    0.7500         0         0
         0    1.0000         0         0
```

```
% 3: TLTD to post-Trailing Leg Touchdown (pTLTD)
T_TLTD_pTLTD_u = probs_u{10:13, 15:25};
T_TLTD_pTLTD_u = convertTransMat(T_TLTD_pTLTD_u)
```

```
T_TLTD_pTLTD_u = 4×11
         0    0.0667         0         0    0.1333    0.3333         0    0.0667 ···
         0    0.2222    0.1111         0         0         0         0         0
    0.2500    0.7500         0         0         0         0         0         0
         0         0    0.5000         0         0         0         0         0
```

```
T_TLTD_pTLTD_b = probs_b{10:13, 15:25};
T_TLTD_pTLTD_b = convertTransMat(T_TLTD_pTLTD_b)
```

```
T_TLTD_pTLTD_b = 4×11
    0.2222    0.4444    0.2222         0         0    0.1111         0         0 ···
    0.3077    0.2308    0.1538    0.0769         0    0.1538         0         0
         0         0    0.3333         0    0.1667    0.1667    0.1667         0
         0         0         0         0         0         0         0         0
```

```
% 4: pTLTD to Outcome (OC)
T_pTLTD_OC_u = probs_u{14:24, 26:28};
T_pTLTD_OC_u = convertTransMat(T_pTLTD_OC_u)
```

```
T_pTLTD_OC_u = 11×3
    1.0000         0         0
    1.0000         0         0
    1.0000         0         0
         0         0         0
    1.0000         0         0
    1.0000         0         0
         0         0         0
         0    1.0000         0
         0         0         0
    0.9167    0.0833         0
      ⋮
```

```
T_pTLTD_OC_b = probs_b{14:24, 26:28};
T_pTLTD_OC_b = convertTransMat(T_pTLTD_OC_b)
```

```
T_pTLTD_OC_b = 11×3
    0.8333         0    0.1667
    0.4286    0.1429    0.4286
    0.8333         0    0.1667
    1.0000         0         0
    1.0000         0         0
    1.0000         0         0
    1.0000         0         0
         0         0         0
    1.0000         0         0
         0         0    1.0000
```

⋮

Check that the probabilities sum to 1

```
sum(T_TR_SI_u)
```

```
ans = 1
```

```
sum(T_TR_SI_b)
```

```
ans = 1.0000
```

```
sum(T_SI_TLTD_u, 2)
```

```
ans = 8×1
    1.0000
    1.0000
    1.0000
         0
         0
    1.0000
         0
         0
```

```
sum(T_SI_TLTD_b, 2)
```

```
ans = 8×1
     0
     1
     1
     1
     1
     1
     1
     1
```

```
sum(T_TLTD_pTLTD_u, 2)
```

```
ans = 4×1
     1
     1
     1
     1
```

```
sum(T_TLTD_pTLTD_b, 2)
```

```
ans = 4×1
    1.0000
    1.0000
    1.0000
         0
```

```
sum(T_pTLTD_OC_u, 2)
```

```
ans = 11×1
     1
     1
     1
     0
     1
```

```
     1
     0
     1
     0
     1
     ⋮
```

```
sum(T_pTLTD_OC_b, 2)
```

```
ans = 11×1
     1
     1
     1
     1
     1
     1
     1
     0
     1
     1
     ⋮
```

Reconstruct Global Transition Matrix for Unilateral Slips (a 27x27 square matrix)

There are 27 total states identified in the graphical model

```
Y_u = probs_u{1:24,3:28};
Y_u = replace(Y_u, 'NA', '0'); % change NA values to 0
Y_u = convertTransMat(Y_u);

Y_b = probs_b{1:24,3:28};
Y_b = replace(Y_b, 'NA', '0'); % change NA values to 0
Y_b = convertTransMat(Y_b);

% Include all 27 states as both rows and columns in the Global Transition
% Matrix by adding an initial column for trigger, and appending 3 rows for
% fall outcomes
triggerColumn = zeros(24,1);
Y_TriggerAdded_u = [triggerColumn, Y_u];
Y_TriggerAdded_b = [triggerColumn, Y_b];

outcomeRows = zeros(3,27);
Y_Global_u = [Y_TriggerAdded_u; outcomeRows]
```

```
Y_Global_u = 27×27
         0    0.1667    0.4667    0.3000         0         0    0.0667         0 ⋯
         0         0         0         0         0         0         0         0
         0         0         0         0         0         0         0         0
         0         0         0         0         0         0         0         0
         0         0         0         0         0         0         0         0
         0         0         0         0         0         0         0         0
         0         0         0         0         0         0         0         0
         0         0         0         0         0         0         0         0
         0         0         0         0         0         0         0         0
```

```
         0         0         0         0         0         0         0         0
      ⋮
```

```
Y_Global_b = [Y_TriggerAdded_b; outcomeRows]
```

```
Y_Global_b = 27×27
         0         0    0.1786    0.0357    0.2857    0.1071    0.2143    0.1429 ···
         0         0         0         0         0         0         0         0
         0         0         0         0         0         0         0         0
         0         0         0         0         0         0         0         0
         0         0         0         0         0         0         0         0
         0         0         0         0         0         0         0         0
         0         0         0         0         0         0         0         0
         0         0         0         0         0         0         0         0
         0         0         0         0         0         0         0         0
         0         0         0         0         0         0         0         0
      ⋮
```

Calculate the probability of each state occuring, $p(x_i)$

```
% On each trial, the probability of trigger is 1
p_TR_u = 1;
p_TR_b = 1;

% The probability of each slip initiation state is the probability of the
% trigger state multiplied by the transition probability to each slip
% initation state. This is simply T_TR_SI.
p_SI_u = T_TR_SI_u;
p_SI_b = T_TR_SI_b;

% The probability for each TLTD state is calculated as the probability of
% each SI state, p_xi(2:9), pre-multiplied by the transition matrix T_SI_TLTD
p_TLTD_u = p_SI_u*T_SI_TLTD_u;
p_TLTD_b = p_SI_b*T_SI_TLTD_b;

% The same process for pTLTD...
p_pTLTD_u = p_TLTD_u*T_TLTD_pTLTD_u;
p_pTLTD_b = p_TLTD_b*T_TLTD_pTLTD_b;

% and for OC
p_OC_u = p_pTLTD_u*T_pTLTD_OC_u
```

```
p_OC_u = 1×3
    0.9333    0.0667         0
```

```
p_OC_b = p_pTLTD_b*T_pTLTD_OC_b
```

```
p_OC_b = 1×3
    0.7500    0.0357    0.2143
```

```
% The outcome probabilities multipled by 30 should give us n for each
% outcome state
```

```
n_OC_u = p_OC_u*30
```

```
n_OC_u = 1×3
    28     2     0
```

```
n_OC_b = p_OC_b*28
```

```
n_OC_b = 1×3
   21.0000    1.0000    6.0000
```

```
% State probabilities for all 27 states
p_Global_u = [p_TR_u p_SI_u p_TLTD_u p_pTLTD_u p_OC_u]
```

```
p_Global_u = 1×27
    1.0000    0.1667    0.4667    0.3000         0         0    0.0667         0 ···
```

```
p_Global_b = [p_TR_b p_SI_b p_TLTD_b p_pTLTD_b p_OC_b]
```

```
p_Global_b = 1×27
    1.0000         0    0.1786    0.0357    0.2857    0.1071    0.2143    0.1429 ···
```

Calculate I(X), the information in each transition from state i to j $\log_2 \frac{1}{P_{i,j}}$

```
I_u = log2(1./Y_Global_u)
```

```
I_u = 27×27
       Inf    2.5850    1.0995    1.7370       Inf       Inf    3.9069       Inf ···
       Inf       Inf       Inf       Inf       Inf       Inf       Inf       Inf
       Inf       Inf       Inf       Inf       Inf       Inf       Inf       Inf
       Inf       Inf       Inf       Inf       Inf       Inf       Inf       Inf
       Inf       Inf       Inf       Inf       Inf       Inf       Inf       Inf
       Inf       Inf       Inf       Inf       Inf       Inf       Inf       Inf
       Inf       Inf       Inf       Inf       Inf       Inf       Inf       Inf
       Inf       Inf       Inf       Inf       Inf       Inf       Inf       Inf
       Inf       Inf       Inf       Inf       Inf       Inf       Inf       Inf
       Inf       Inf       Inf       Inf       Inf       Inf       Inf       Inf
     ⋮
```

```
I_b = log2(1./Y_Global_b)
```

```
I_b = 27×27
       Inf       Inf    2.4854    4.8074    1.8074    3.2224    2.2224    2.8074 ···
       Inf       Inf       Inf       Inf       Inf       Inf       Inf       Inf
       Inf       Inf       Inf       Inf       Inf       Inf       Inf       Inf
       Inf       Inf       Inf       Inf       Inf       Inf       Inf       Inf
       Inf       Inf       Inf       Inf       Inf       Inf       Inf       Inf
       Inf       Inf       Inf       Inf       Inf       Inf       Inf       Inf
       Inf       Inf       Inf       Inf       Inf       Inf       Inf       Inf
       Inf       Inf       Inf       Inf       Inf       Inf       Inf       Inf
       Inf       Inf       Inf       Inf       Inf       Inf       Inf       Inf
       Inf       Inf       Inf       Inf       Inf       Inf       Inf       Inf
     ⋮
```

Calculate the Entropy of the Global Transition Matrix for Unilateral Slips

$$H(Y) = E(I(X)) = \sum_{i,j} p(x_i) P_{i,j} \log_2 \frac{1}{P_{i,j}}$$

```
% Using the entropy calculation as written in "Method for Assessing Slip
% Diversity" word document
p_Global_repeat_u = repmat(p_Global_u, 27, 1)';
RHS_u = p_Global_repeat_u .* Y_Global_u .* I_u;
RHS_u(isnan(RHS_u)) = 0;
H_u = sum(RHS_u, 'all')
```

```
H_u = 5.0220
```

```
p_Global_repeat_b = repmat(p_Global_b, 27, 1)';
RHS_b = p_Global_repeat_b .* Y_Global_b .* I_b;
RHS_b(isnan(RHS_b)) = 0;
H_b = sum(RHS_b, 'all')
```

```
H_b = 6.0992
```

Function to Extract and Convert Transition Matrices

```
function T_output = convertTransMat(T_input)
for i = 1:numel(T_input)
    T_input{i} = str2double(T_input{i});
end
T_output = cell2mat(T_input);
end
```
