# Supplementary material for: Higher fall rates and broader kinematic diversity in bilateral versus unilateral unconstrained slips
Source: PLoS One. 2025 Aug 7;20(8):e0328900. doi: 10.1371/journal.pone.0328900 (PMC12331078; doi:10.1371/journal.pone.0328900)
Supplement: S1 Fig — (PDF) [file pone.0328900.s005.pdf]

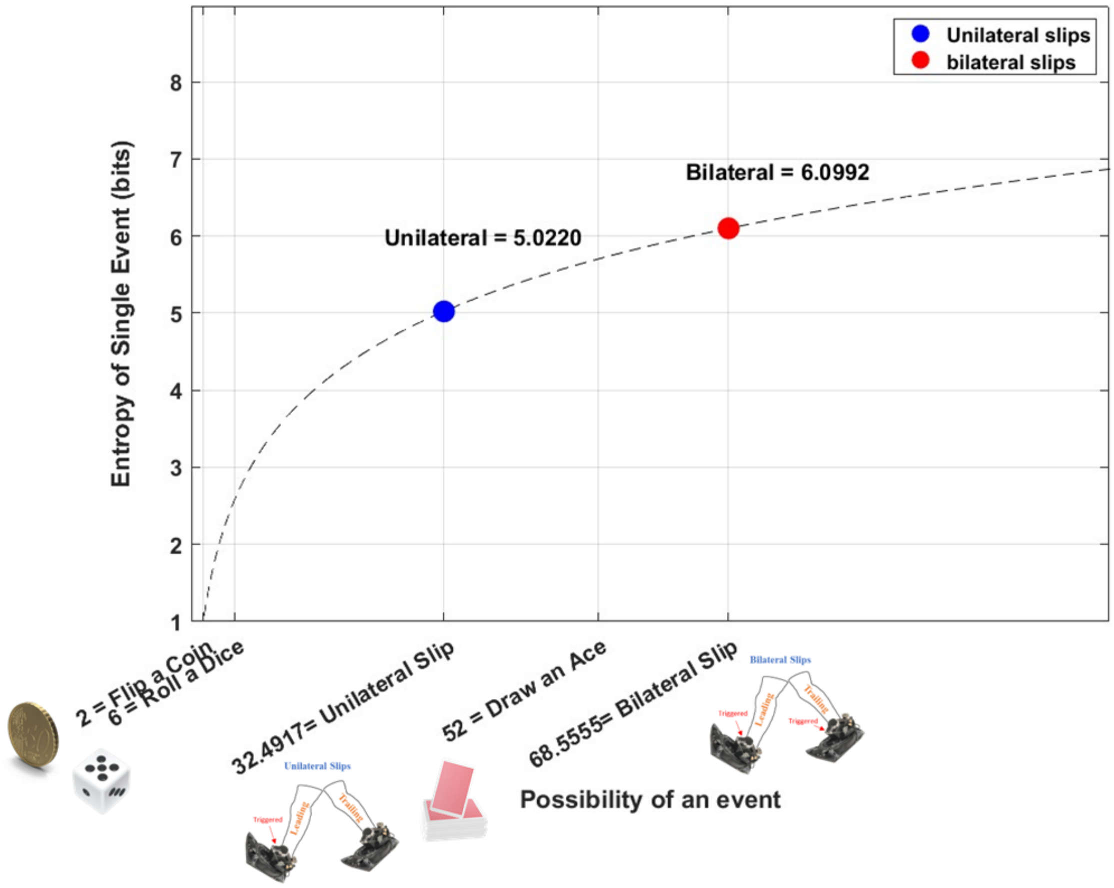

Supplementary Fig 1. Differences in the Entropy and the Possibility of a Single Event between Unilateral and Bilateral Slips. Flipping a coin results in "2" possible events (heads/tails) and an entropy of "1". Rolling a dice results in "6" possible events (1 to 6) and an entropy of "2.5850". Unilateral slips results in "32.4917" possible events and an entropy of "5.0220". Drawing a card from a card deck results in "52" possible events and an entropy of "5.7004". Bilateral slips results in "68.5555" possible events and an entropy of "6.0992".
